# Evolution and Diversity of TGF-β Pathways are Linked with Novel Developmental and Behavioral Traits

**DOI:** 10.1093/molbev/msac252

**Published:** 2022-12-05

**Authors:** Wen-Sui Lo, Marianne Roca, Mohannad Dardiry, Marisa Mackie, Gabi Eberhardt, Hanh Witte, Ray Hong, Ralf J Sommer, James W Lightfoot

**Affiliations:** Department for Integrative Evolutionary Biology, Max-Planck Institute for Biology Tübingen, Max-Planck Ring 9, 72076 Tübingen, Germany; Max Planck Research Group Genetics of Behavior, Max Planck Institute for Neurobiology of Behavior—Caesar, Ludwig-Erhard-Allee 2, 53175, Bonn, Germany; Department for Integrative Evolutionary Biology, Max-Planck Institute for Biology Tübingen, Max-Planck Ring 9, 72076 Tübingen, Germany; Department of Biology, California State University, Northridge, CA; Department for Integrative Evolutionary Biology, Max-Planck Institute for Biology Tübingen, Max-Planck Ring 9, 72076 Tübingen, Germany; Department for Integrative Evolutionary Biology, Max-Planck Institute for Biology Tübingen, Max-Planck Ring 9, 72076 Tübingen, Germany; Department of Biology, California State University, Northridge, CA; Department for Integrative Evolutionary Biology, Max-Planck Institute for Biology Tübingen, Max-Planck Ring 9, 72076 Tübingen, Germany; Max Planck Research Group Genetics of Behavior, Max Planck Institute for Neurobiology of Behavior—Caesar, Ludwig-Erhard-Allee 2, 53175, Bonn, Germany

**Keywords:** TGF-β signaling, *Pristionchus pacificus*, *Caenorhabditis elegans*, developmental systems drift, dauer, signaling pathway evolution, phenotypic plasticity, behavior, kin-recognition

## Abstract

Transforming growth factor-β (TGF-β) signaling is essential for numerous biologic functions. It is a highly conserved pathway found in all metazoans including the nematode *Caenorhabditis elegans*, which has also been pivotal in identifying many components. Utilizing a comparative evolutionary approach, we explored TGF-β signaling in nine nematode species and revealed striking variability in TGF-β gene frequency across the lineage. Of the species analyzed, gene duplications in the DAF-7 pathway appear common with the greatest disparity observed in *Pristionchus pacificus.* Specifically, multiple paralogues of *daf-3*, *daf-4* and *daf-7* were detected. To investigate this additional diversity, we induced mutations in 22 TGF-β components and generated corresponding double, triple, and quadruple mutants revealing both conservation and diversification in function. Although the DBL-1 pathway regulating body morphology appears highly conserved, the DAF-7 pathway exhibits functional divergence, notably in some aspects of dauer formation. Furthermore, the formation of the phenotypically plastic mouth in *P. pacificus* is partially influenced through TGF-β with the strongest effect in *Ppa-tag-68.* This appears important for numerous processes in *P. pacificus* but has no known function in *C. elegans.* Finally, we observe behavioral differences in TGF-β mutants including in chemosensation and the establishment of the *P. pacificus* kin-recognition signal. Thus, TGF-β signaling in nematodes represents a stochastic genetic network capable of generating novel functions through the duplication and deletion of associated genes.

## Introduction

Signaling pathways mediate fundamental roles ranging from development to behavior. The Transforming growth factor-β (TGF-β) family is one of the most multifaceted and versatile metazoan signaling pathways and is essential for a wide range of functions. These include roles in development, immunity, cell fate, aging, homeostasis, tissue repair, and metabolism (Massagué 2012; Weiss and Attisano 2013; Batlle and Massagué 2019; Tominaga and Suzuki 2019). Accordingly, various heritable disorders in humans are also attributed to mutations in the TGF-β machinery and additionally, with its involvement in the inhibition of cell-cycle progression and induction of apoptosis, its dysregulation is also implicated in the development of several forms of cancer (Massagué et al. 2000). TGF-β members are a family of secreted peptide growth factors, which act as ligands to recruit and assemble receptor complexes. These signaling complexes in turn activate the Smad transcription factors to regulate gene expression. More specifically, the TGF-β ligands bind to cell surface serine/threonine kinase receptors belonging to two different groups (types I and II). Upon the binding of the TGF-β ligand, a hetrotetrameric complex is recruited consisting of both types of receptors with type II receptors phosphorylating and activating type I receptors. The activation of type I receptors in turn act upon another class of proteins called the Smads, phosphorylating the R-Smads (receptor regulated), which recruit Co-Smads (common mediator) to accumulate in the nucleus and regulate transcription (Massagué 2012). An additional class of I-Smads (inhibitory) together with other extracellular regulators also helps control Smad activation. Additionally, TGF-β receptors also activate non-Smad signaling pathways which further contributes to the complexity of the signal induced (Zhang 2017). Therefore, there exists the potential for a large diversity of functional regulation through TGF-β signaling as each ligand can act on several receptor types and cell-type-specific expression of receptors is also observed.

TGF-β signaling is an evolutionarily ancient pathway. In humans, 33 members of the TGF-β family have been identified including seven type I receptors and five type II receptors (Heldin and Moustakas 2016; Morikawa et al. 2016), components have also been found in the most basal of metazoan genomes (Huminiecki et al. 2009; Redmond and McLysaght 2021). This includes the identification of eight TGF-β ligands and five receptors in marine sponges (Srivastava et al. 2010; Riesgo et al. 2014), and in the Placozoan, *Trichoplax adhaerens*, at least four TGF-β receptors and four Smads are present (Srivastava et al. 2008; Huminiecki et al. 2009). Additionally, in model organisms including *Drosophila* and the nematode *Caenorhabditis elegans*, seven and five TGF-β family members have been detected, respectively. Moreover, both these invertebrate model organisms pioneered the identification of many genes involved in the TGF-β signaling pathway. This includes the identification of the first serine/threonine kinase type I receptor *daf-1* in *C. elegans.* Mutations in this gene cause worms to erroneously enter the long-lived stress resistant dauer life cycle stage (*daf*: abnormal *da*uer *f*ormation), normally utilized to avoid harsh environmental conditions (Georgi et al. 1990). Additionally, numerous Smad proteins were discovered in these organisms, which derive their name from a combination of the *C. elegans* small body phenotype associated with these mutants (*sma*) (Estevez et al. 1993; Savage et al. 1996), and *Drosophila* mutants identified in a genetic enhancer screen –*mothers against Decapentaplegic (Mad)* (Raftery et al. 1995; Sekelsky et al. 1995; Moses et al. 2016).

In *C. elegans*, the five TGF-β signaling ligands are *dbl-1*, *daf-7*, *unc-129*, *tig-2*, and *tig-3* with many of these signaling pathways having well-characterized downstream components and functions (Patterson and Padgett 2000). The DBL-1 pathway was first identified through the small body size (*sma*) and abnormal male tail (*mab*) observed in loss of function mutants. DBL-1 is expressed in several neurons including head sensory neurons (Morita et al. 1999; Suzuki et al. 1999; Morita et al. 2002) from where it signals the type I/II receptor pair of SMA-6 and DAF-4. These in turn, act on SMA-2, SMA-3, and SMA-4 to induce transcription and regulate a panoply of phenotypes including body size (Morita et al. 2002; Dineen and Gaudet 2014), male tail development (Savage et al. 1996), immunity (Zhang and Zhang 2009), aging (Luo et al. 2010), lipid accumulation (Clark et al. 2018), and neuronal functions such as neural plasticity in olfactory aversive learning (Vashlishan et al. 2008; Zhang and Zhang 2012). In the DAF-7 signaling pathway, many components were initially identified due to the improper entry or exit from the dauer life cycle stage. DAF-7 is expressed in a small subset of sensory neurons, which can vary depending on environmental conditions (Ren et al. 1996; Meisel et al. 2014). It signals the type I/II receptors DAF-1/DAF-4 with this signal transduced by the Smads of DAF-8 and DAF-14. In addition to regulating dauer formation, DAF-7 signaling governs an array of processes including fat storage (Greer et al. 2008), behavioral activities associated with feeding (You et al. 2008), male-specific behaviors (Hilbert and Kim 2017), and also aging, which occurs through mediation of the insulin signaling pathway (Shaw et al. 2007). UNC-129 represents a more atypical TGF-β signaling ligand with no known type I/II receptors and is thought to regulate the migration of motor neurons (Colavita et al. 1998; MacNeil et al. 2009). A similar function in motor neuron migration has also recently been described for TIG-2 and TIG-3 (Gumienny and Savage-Dunn 2013; Savage-Dunn and Padgett 2017; Baltaci et al. 2022).

Most of our understanding of cell signaling networks in nematodes comes from research on *C. elegans.* However, the phylum Nematoda provides a powerful system to study the evolution of signaling pathways (Gilabert et al. 2016). For example, previous studies, utilizing *C. elegans* and a comparative nematode system *Pristionchus pacificus,* have highlighted how conserved morphologic structures can be generated by diverse signaling networks through a phenomenon known as evolutionary systems drift. Specifically, *C. elegans* vulva formation is regulated through EGF-RAS signaling, whereas in *P. pacificus*, a morphologically homologous vulva structure is instead generated through Wnt signaling (Wang and Sommer 2011; Sommer 2012). For TGF-β signaling, comparative studies investigating its evolution across nematodes have often focused on its role in the regulation of dauer formation. This is due to the similarities between the dauer stage in free-living nematodes and the infective larvae of many parasitic nematode species (Viney et al. 2005; Gilabert et al. 2016). As such, several TGF-β family members have been identified in parasitic nematodes, although functional studies are problematic due to the complex life cycles found in these organisms (McSorley et al. 2009; Ogawa et al. 2009; Crook et al. 2010; Ma et al. 2019; Yang et al. 2019; He et al. 2020). Interestingly, a recent study in *P. pacificus* demonstrated that the *Ppa-daf-7* pathway is necessary for bacterial induced gut–brain communication, which in turn enhanced numerous developmental phenotypes (Lo et al. 2022). Moreover, multiple *daf-7*-type signaling ligands have been identified suggesting a previously unknown element to TGF-β signaling including the possibility of its neofunctionalization outside of the canonical *C. elegans* pathway.

In this study, we reveal that the TGF-β signaling pathway is highly divergent across the nematode phylum. By focusing on comparative studies between *C. elegans* and *P. pacificus*, we reveal this divergence also corresponds to functional differences including in the key signaling ligands *Ppa-tig-2*, *Ppa-unc-129*, and *Ppa-daf-7*. Within the *Ppa-daf-7* signaling pathway, we find the most striking functional variance including the regulation of *P. pacificus* developmental processes determining dauer formation, mouth structure as well as in behaviors such as chemosensation and kin-recognition. Thus, TGF-β signaling is an important pathway for the evolution of diverse and novel traits by regulating distinct developmental and behavioral properties across species.

## Results

### TGF-β Signaling Pathways are Highly Divergent Across Nematode Evolution

**Fig. 1.**
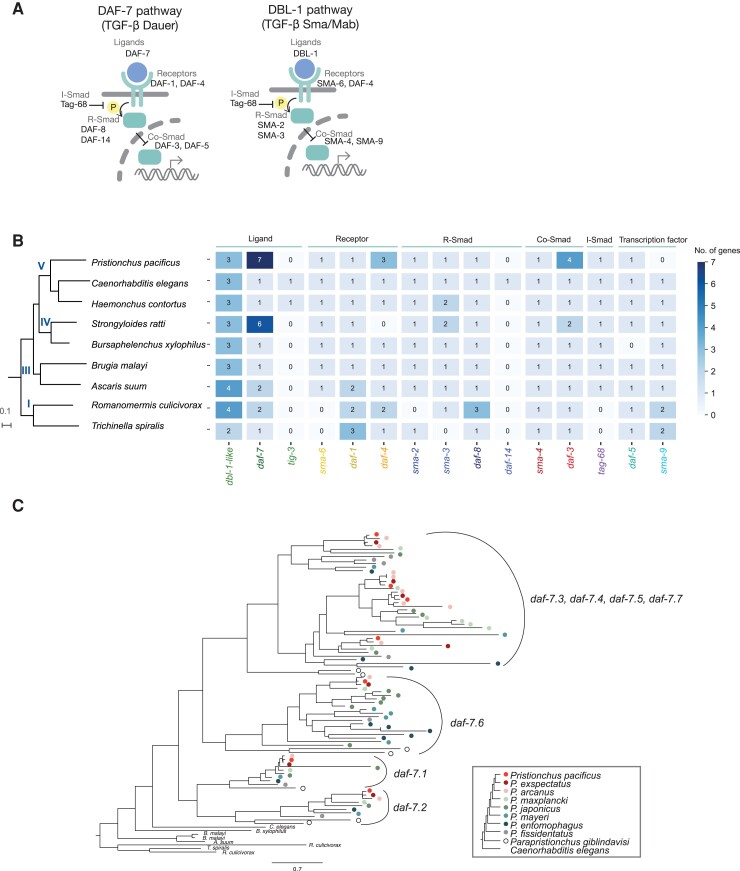
The evolution of the TGF-β signaling pathway in nematodes. (*A*) Schematic representation of the two main TGF-β signaling pathways and their main components in nematodes. (*B*) A phylogeny of nine nematodes species (left), and the number of orthologous genes in each species. The TGF-β ligands *dbl-1*, *tig-2*, and *unc-129* were assigned to the same orthogroup, and were referred to as *dbl-1* like. (*C*) The gene tree of *daf-7* genes in the genus *Pristionchus.* An unscaled phylogeny in the legend shows the relationship of the genus *Pristionchus* (left).

To investigate the evolution of the TGF-β signaling pathway in nematodes (fig. 1*A*), we first performed a comparative genomic analysis using nine representative nematode species. TGF-β components were identified based on their sequence similarity with *C. elegans.* We found that in comparison with the DBL-1 pathway, the copy number of genes involved in the DAF-7 pathway is variable across both parasitic and free-living nematodes (fig. 1*B*). Additionally, of the species examined, a homolog of *tig-3* was only detected in *C. elegans* and *Haemonchus contortus*, whereas *daf-14* was identified in *C. elegans* alone. Thus, *tig-3* and *daf-14* were likely acquired relatively late in the phylum Nematoda and are restricted to a few species in clade V. Surprisingly, in several species, multiple gene copies were detected of the TGF-β signaling ligand *daf-7*, type I receptor *daf-1*, type II receptor *daf-4*, R-Smads *daf-8*, and Co-Smad *daf-3*, suggesting that gene duplications may be a common phenomenon in the nematode DAF-7 pathway.

In *P. pacificus*, we detected a particularly extreme example of these duplication events with seven copies of *daf-7* found throughout its genome. Thus, the relative simplicity of *daf-7* ligands in *C. elegans* represents a feature that might have benefited its functional characterization. Taking advantage of the available genome assembly data across the genus *Pristionchus* (Prabh et al. 2018), we next investigated the evolution of the *daf-7* genes in more detail. The phylogeny of *daf-7* genes from eight *Pristionchus* species and its basal relative *Parapristionchus giblindavisi* indicates that this gene family expansion has resulted from multiple duplication events throughout the genus (fig. 1*C*). *Ppa*-*daf-7.1* and *Ppa*-*daf-7.2* are closely related to the *daf-7* gene of other nematodes and additionally, the shorter branch lengths of the *daf-7.1* genes suggest they are likely the ancestral copies. In the further derived *Ppa*-*daf-7.6* clade, the topologies are generally consistent with the known species relationship. However, species-specific gene duplication events are observed in *Pristionchus japonicus*, *Pristionchus mayeri*, and *Pristionchus entomophagus*. Finally, the *Ppa*-*daf-7.3*, *Ppa*-*daf-7.4*, *Ppa*-*daf-7.5*, and *Ppa*-*daf-7.7* are derived from recent gene duplication events. Together, these data suggest that the *daf-7* genes in *Pristionchus* are particularly rapidly evolving.

### TGF-β Signaling Mutants Induce Body Morphology Defects

With our phylogenetic analysis revealing exceptional diversity in the TGF-β pathway across the nematode phylum, we decided to investigate this variation in gene frequency via functional studies. Therefore, we focused our attention on *P. pacificus* as multiple copies of many TGF-β components were detected and an abundance of genetic and molecular tools are available (Witte et al. 2015; Rödelsperger et al. 2017; Han et al. 2020; Nakayama et al. 2020). We first utilized CRISPR/Cas9 to generate putative null mutants in many of the TGF-β pathway components in *P. pacificus*. In total, we targeted 22 TGF-β related genes including all of the TGF-β signaling ligands, *Ppa-dbl-1*, *Ppa-unc-129*, *Ppa-tig-2*, and its seven *Ppa-daf-7* copies. Furthermore, as the *Ppa-daf-7* copies fall into phylogenetic groupings, we generated a *Ppa-daf-7.1; daf-7.2* double mutant and a *daf-7.3; daf-7.4; daf-7.5; daf-7.7* quadruple mutant accordingly. There is no identifiable *tig-3* homologue in *P. pacificus*. We generated mutants in the type I receptor *Ppa-daf-1* and the three copies of the type II receptor *Ppa-daf-4*, including constructing an associated triple mutant. Additionally, we produced mutations in the R-Smads *Ppa-sma-2* and *Ppa-daf-8*. We also generated mutants in the four copies of the Co-Smad *Ppa-daf-3*, as well as generating the corresponding quadruple mutant. Finally, we induced mutations in the I-Smad *Ppa-tag-68* and the transcription factor *Ppa-daf-5.* In the majority of cases, we were successful in producing at least two alleles for each mutant for functional analysis (supplementary figs. S1 and S2, and tables S1 and S2, Supplementary Material online).

**Fig. 2.**
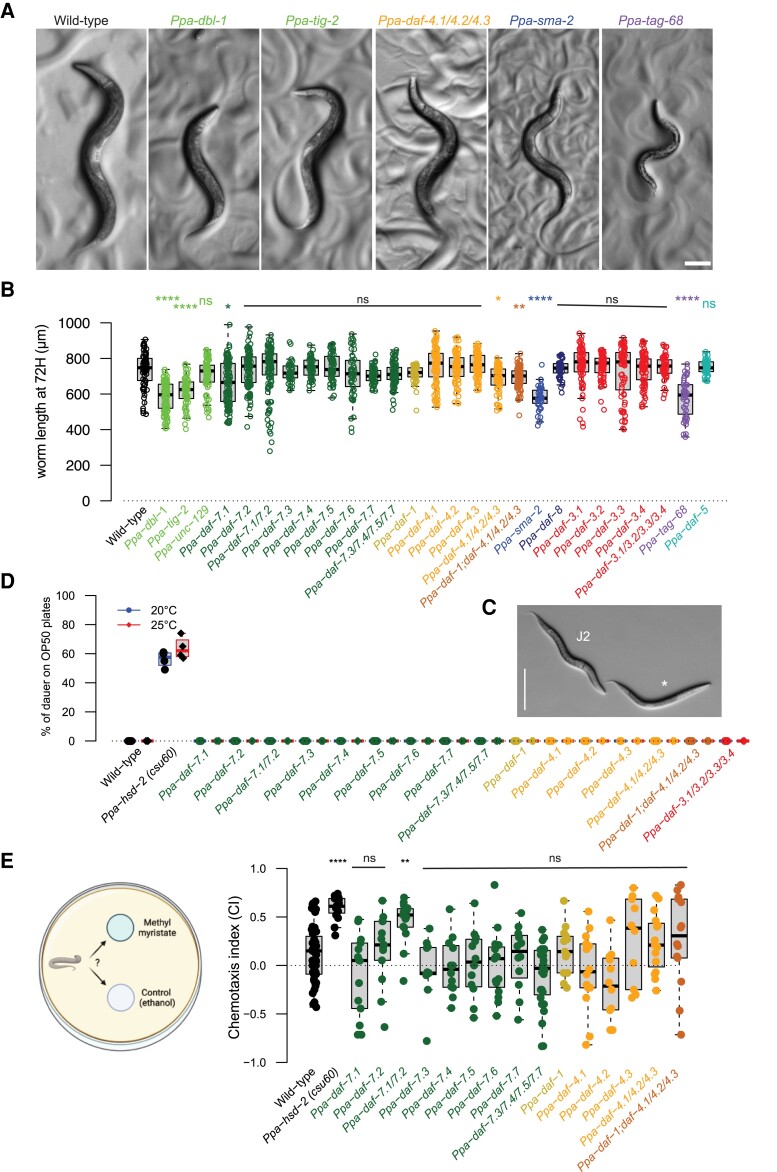
Conservation and diversification of canonical TGF-β mutant phenotypes. (*A*) Representative images of TGF-β mutants with morphologic body size defects. Scale bar = 100 µm. (*B*) Length of TGF-β mutants after 72 h growth and fed on OP50. Data are represented as a box plot with several mutants showing statistically significant body length abnormalities (Wilcoxon Mann–Whitney test with Bonferroni correction; *P ≤ 0.05, **P ≤ 0.01, ****P ≤ 0.0001). A minimum of 40 animals were measured for each mutant. (*C*) Image of a *Pristionchus pacificus* J2 larvae alongside a dauer (*) larvae showing their morphologic difference. Scale bar = 100 µm. (*D*) Number of dauer stage animals observed under two different culture conditions. *Ppa-hsd-2(csu60)* is a control for *daf-c* phenotype. (*E*) Mean adult *Ppa-daf-7* mutants’ response to 1% MM. *Ppa-hsd-2(csu60)* is a control for enhanced attraction to MM (Carstensen et al. 2021). The *Ppa-daf-7.1; Ppa-daf-7.2* double mutant shows significantly higher attraction to 1% MM compared with wild type (Wilcoxon Mann–Whitney test with Bonferroni correction; **P ≤ 0.01, ****P ≤ 0.0001). At least ten assays were performed for all conditions. ns, non-significant.

As many developmental phenotypes including defects in body morphology and size are associated with the TGF-β pathway in *C. elegans*, we first analyzed our *P. pacificus* mutants for similar deficiencies (fig. 2*A*and*B*). In *C. elegans*, body size mutants include *Cel-dbl-1*, *Cel-sma-2*, and *Cel-daf-4* are all ∼70% smaller by volume than wild type (Flemming et al. 2000; Maduzia et al. 2002; Morita et al. 2002). Similar to the *C. elegans* mutants, both *Ppa-dbl-1* and *Ppa-sma-2* are morphologically smaller in comparison with wild-type worms (20% shorter and 21% shorter, respectively). Additionally, the *Ppa-daf-4.; daf-4.2; daf-4.3* triple mutant was also smaller (6.7%) demonstrating functional redundancy between these components. However, differing from *C. elegans*, *Ppa-dbl-1* appears *dumpy* rather than *small* and additionally morphological defects are evident from the larval stage in all these mutants, whereas they become apparent in later developmental stages in *C. elegans* (supplementary fig. S3, Supplementary Material online). This may be due to differences in larval development as *P. pacificus* hatches at the second larval stage compared with *C. elegans* which hatches at the first larval stage. Unexpectedly, we also observed morphological defects in *Ppa-tag-68* which are small (21% shorter) and sickly, whereas *Ppa-tig-2* mutants are 16% shorter. Finally, *Cel-unc-129* is thought to act as a guidance cue for axon migration and is therefore essential for nervous system function. As such, *Cel-unc-129* mutants appear *uncoordinated* (*unc*) and unable to move effectively. However, in *Ppa-unc-129*, we observe no *unc* phenotype and no alterations in body morphology indicating divergence in at least some aspects of this gene function between species (Supplementary movie 1, Supplementary Material online).

### Dauer Formation may be Independent of TGF-β Signaling

In *C. elegans*, many TGF-β family components were identified through screens for animals defective in dauer formation. Dauer larvae provide an alternative larval stage, which is highly stress resistant and aids survival during adverse conditions such as low nutrient availability and other environmental stressors. Animals can remain in the dauer stage until external conditions improve, upon which they can then continue their development to adult nematodes. In *C. elegans*, mutations in the TGF-β pathway can result in either dauer-defective (*daf-d*) phenotypes including in *Cel-daf-3* and *Cel-daf-5* or dauer-constitutive (*daf-c*) phenotypes such as *Cel-daf-7* (Gumienny and Savage-Dunn 2013). Therefore, we assessed the *P. pacificus* TGF-β signaling mutants to observe any *daf-c* phenotypes associated with erroneous dauer entry. Surprisingly, in all of the *P. pacificus* TGF-β mutants tested, dauer regulation appeared unaffected, with dauer formation occurring only after starvation, and no constitutive dauer larvae observed on food either at 20 °C or 25 °C (fig. 2*C*and*D*). Furthermore, in *C. elegans*, developmental dependent expression of *Cel-daf-7* is observed with expression evident in the amphid neurons of the L1 and L2 larvae preceding the dauer stage but not in the dauer larvae itself (Ren et al. 1996). We therefore utilized previously generated *Ppa-daf-7.1* and *Ppa-daf-7.2* transcriptional reporters (Lo et al. 2022) to investigate any similar developmental restricted expression patterns in *P. pacificus*. However, both reporters showed robust and consistent expression in amphid neurons in both well-fed J2 and starvation-induced dauer larvae in *P. pacificus* indicating distinct regulation of *daf-7* between species (supplementary fig. S4, Supplementary Material online). Thus, our results imply dauer regulation via TGF-β may have been lost in the *P. pacificus* evolutionary lineage or alternatively, dauer regulation may have been co-opted during the evolution of *C. elegans*. These possibilities may be clarified further by the creation of a full *Ppa-daf-7* null mutant consisting of mutations in all seven *Ppa-daf-7* paralogues. Importantly, due to the similarities between the dauer stage in free-living nematodes and the infective larvae in parasitic nematode species, the presumed association of TGF-β signaling with dauer formation has formed the basis for substantial parasitic research as a potential target to understand their infectivity (McSorley et al. 2009; Ogawa et al. 2009; Crook et al. 2010; Ma et al. 2019; Yang et al. 2019; He et al. 2020). Therefore, functional studies in other nematode species will be essential to distinguish between these possibilities, and further inform research on parasitic nematodes.

### *Ppa-daf-7* Paralogues are Required for Aspects of Chemosensation

Previous studies in *C. elegans* have found that in addition to its role in dauer formation, *Cel-daf-7* establishes chemosensory receptor gene expression (Nolan et al. 2002) which has subsequently been shown to be involved in establishing an aversive behavioral response to pathogens (de Bono et al. 2002; Meisel et al. 2014). Furthermore, the constitutive dauer larvae of *Cel-daf-7* mutants show significantly lower odor attraction than adults (Vertiz et al. 2021). As *P. pacificus* is frequently found associated with scarab beetles, it has a strong response to several pheromones generated from potential insect host species (Cinkornpumin et al. 2014; Carstensen et al. 2021). Therefore, we assessed our *Ppa-daf-7* mutants and its canonically associated downstream receptors for odortaxis defects by investigating the previously identified attractive compound, methyl myristate (MM). Here, the *Ppa-daf-7.1; Ppa-daf-7.2* double mutant shows significantly higher attraction to 1% MM compared with wild-type controls indicating environmental sensing defects associated with this pathway (fig. 2*E*). Surprisingly, this phenotype was absent in the downstream receptors likely indicating the existence of other regulatory pathways outside of the canonical *Ppa-daf-4* and *Ppa-daf-1*. Furthermore, as *Cel-daf-7* is also important for aggregation behaviors in *C. elegans* (de Bono et al. 2002; Bendesky et al. 2012), we next investigated additional defects by also assessing aggregation behaviors in *Ppa-daf-7* mutants. Contrary to *C. elegans*, most strains of *P. pacificus* are solitary with aggregation behaviors mostly restricted to a single high altitude adapted clade of *P. pacificus*. Furthermore, this behavior in *P. pacificus* is independent of *Ppa-npr-1* (Moreno et al. 2016, 2017). Similarly, all *Ppa-daf-7* mutant permutations also remained solitary when compared with an aggregating strain reinforcing the distinct mechanistic regulation behind these behaviors between *C. elegans* and *P. pacificus* (supplementary fig. S5, Supplementary Material online). Therefore, *Ppa-daf-7* and its paralogues are important for aspects of environmental sensing in *P. pacificus.* Future work will seek to understand the potential additional complexity generated through the large expansion in *Ppa-daf-7* and identify other potential sensory functions.

### TGF-β Signaling Influences Mouth-Form Fate

**Fig. 3.**
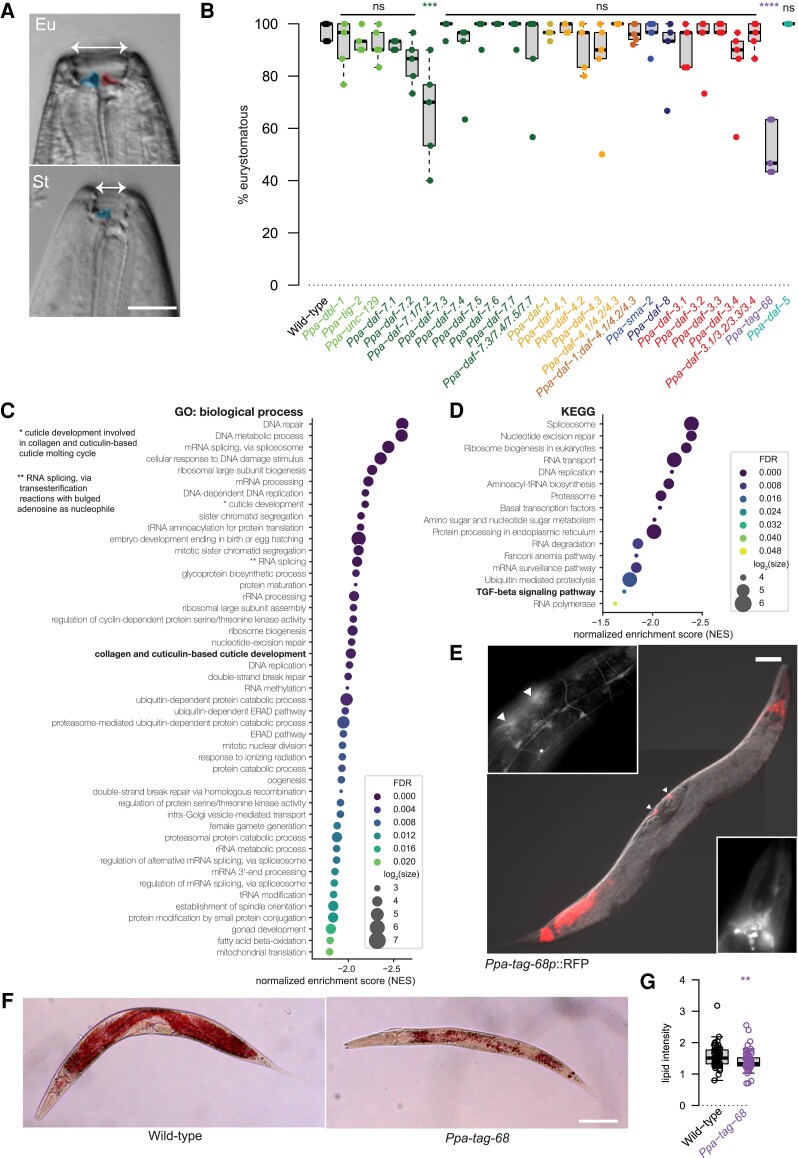
TGF-β mutants are associated with the phenotypically plastic mouth-form fate. (*A*) *Pristionchus pacificus* mouth forms one of two morphs. Eu animals have two teeth with a wide mouth and are omnivores capable of predation on the larvae of other nematodes, whereas St animals have a single tooth with a narrower mouth and are strictly microbivores. Scale bar = 10 µm. Dorsal tooth is left (false colored blue) in both forms and subventral tooth is right (false coloured red) and only present in the Eu morph. (*B*) Mouth-form ratios across all TGF-β mutants generated. Mouth-form frequencies are the mean of five independent replicates. Two-way analysis of variance followed by Bonferroni correction (***P ≤ 0.001, ****P ≤ 0.0001). (*C*) Plots showing normalized enrichment scores of the gene set enrichment analysis in GO biologic process and (*D*) KEGG of *Ppa-tag-68.* The size indicates the number of genes in each gene set. (*E*) Expression pattern of *Ppa-tag-68p*::RFP. Broad expression is observed in many neurons. Top insert shows 3× magnification of the ventral nerve cord (VNC) region and a subset of VNC motor neurons. Bottom insert shows 3× magnification of the head ganglia region. Arrows indicate *P. pacificus* homologues of VC4 and VC5. Scale bar = 50 µm. (*F*) Oil Red O staining to observe lipid storage abundance in wild-type animals compared with *Ppa-tag-68*. Less red staining in *Ppa-tag-68* indicates there is less lipid distribution in this mutant. Scale bar = 100 µm. (*G*) Quantification of Oil Red O lipid staining for at least 64 worms per strain, **P ≤ 0.01. FDR, false discovery rate; ns, non-significant.

Although *P. pacificus* and *C. elegans* share many developmental and behavioral traits, there are also several key morphologic and behavioral differences between these species. Most strikingly, there are differences in mouth morphology as *P. pacificus* possesses teeth-like denticles, which diversify its diet and behaviors to include predating and feeding upon the larvae of other nematodes (Wilecki et al. 2015; Ishita et al. 2021). Furthermore, the *P. pacificus* mouth structure demonstrates additional complexity as it is phenotypically plastic and dimorphic (fig. 3*A*). Animals are therefore capable of forming one of two morphs which are either the two-toothed predatory morph-designated eurystomatous (Eu) or the single-toothed microbivorous morph-designated stenostomatous (St) (Bento et al. 2010). The ratio of these morphs in a given population is variable and dependent on a multitude of environmental and genetic factors (Ragsdale et al. 2013; Werner et al. 2017; Bui et al. 2018; Sieriebriennikov et al. 2020). We therefore, assessed if the expansion in the TGF-β signaling pathway in *P. pacificus* may regulate the mouth-form ratio (fig. 3*B*). A significant change in mouth-form frequency was observed in two mutant strains. In *Ppa-daf-7.1; Ppa-daf-7.2* double mutants, the Eu mouth-form ratio decreased from over 97% in wild-type animals to 66% in the mutant strain. As *Ppa-daf-7.1* and *Ppa-daf-7.2* are expressed in head sensory neurons (Lo et al. 2022) and have previously also been shown to be defective in their response to MM (fig. 2*E*), we hypothesize that the altered mouth-form ratio may also be caused by an inability to sense environmental cues. This is similar to the effect observed in several other environmental sensing defective mutants (Moreno et al. 2019). However, the strongest effect on mouth-form fate was detected in the *P. pacificus* I-Smad homologue *Ppa-tag-68*, in which the ratio of the Eu morph decreased significantly to 52% in *Ppa-tag-68* mutants. In addition to the mouth-form phenotype observed in *Ppa-tag-68*, other previously mentioned defects are evident in this mutant including slow growth and a sickly appearance. Interestingly, in *C. elegans*, *Cel-tag-68* has no known phenotype despite it being the only I-Smad found in the genomes of both nematode species (Gumienny and Savage-Dunn 2013). Therefore, we investigated the *Ppa-tag-68* mutant further to understand its phenotype and function in *P. pacificus*.

With the pleiotropic defects and overall small body size in the *Ppa-tag-68* mutants, we utilized RNA-seq to identify causal pathways behind these phenotypes. From this, we detected deficiencies in a multitude of processes including DNA processing and repair, transcription and protein synthesis pathways, as well as cuticle development and many others which may explain the severity and broad spectrum of *Ppa-tag-68* phenotypes observed (fig. 3*C*and*D* and supplementary fig. S6, Supplementary Material online). Furthermore, a transcriptional reporter line revealed extensive expression throughout the *P. pacificus* nervous system including robust expression in many tail neurons, in several head neurons and in cells homologous to the ventral cord neurons VC4 and VC5 in *C. elegans* (fig. 3*E*). Finally, with the transparent intestinal appearance and the overall small size in *Ppa-tag-68* mutants, we explored a potential link between *Ppa-tag-68* and lipid storage. We observed the accumulation of lipids in both wild type and *Ppa-tag-68* directly using Oil Red O staining, which stains triglycerides and lipoproteins in nematode species including *P. pacificus* (fig. 3*F*and*G*; O’Rourke et al. 2009; Rae et al. 2012). Indeed, comparisons between wild-type animals and *Ppa-tag-68* mutants revealed lower levels of lipid accumulation in the *Ppa-tag-68* intestine. Therefore, *Ppa-tag-68* appears to be essential for many cellular and developmental processes including influencing mouth-form fate in *P. pacificus* and future work must clarify this further through additional targeted mutations.

### Kin-Recognition is Disrupted in *Ppa-daf-7.6* Mutants

**Fig. 4.**
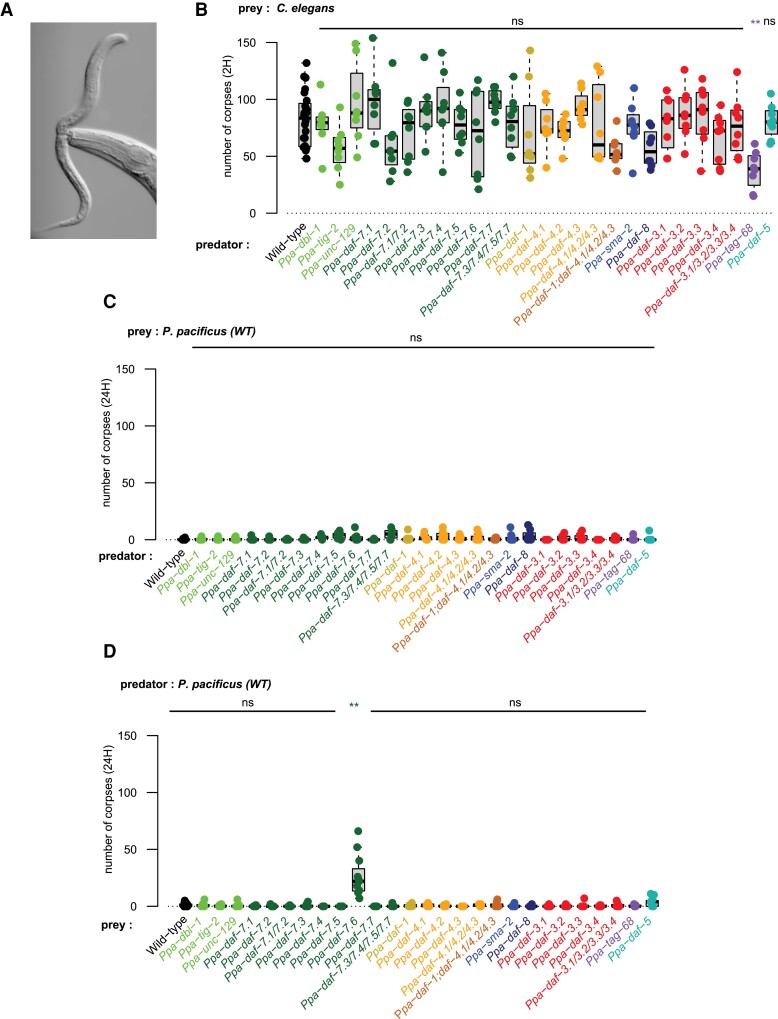
TGF-β mutant are associated with regulation of predation behaviors. (*A*) *Pristionchus pacificus* Eu mouth-form killing a *C. elegans* larvae. (*B*) Corpse assays investigating predation efficiency in all TGF-β mutants generated utilizing 5 young adult mutant *P. pacificus* predators fed on *Caenorhabditis elegans* larvae. Data are represented as box plots of at least eight replicates. Killing defects were only observed in *Ppa-tag-68* mutants (*P* = 0.006, Wilcoxon Mann–Whitney test with Bonferroni correction). (*C*) Corpse assays investigating potential kin-recognition receptor defects in all TGF-β mutants generated. Twenty young adult mutant predators were fed on *P. pacificus* wild-type larvae. Data are represented as box plots of at least eight replicates. No significant defects in kin-recognition receptor function were observed (Wilcoxon Mann–Whitney test with Bonferroni correction). (*D*) Corpse assays investigating potential kin-recognition ligand defects in all TGF-β mutants generated. Young adult wild-type predators were fed on TGF-β mutant larvae. Data are represented as box plots of at least eight replicates. Significant defects in kin-recognition ligand function were observed in *Ppa-daf-7.6* (*P* = 0.003, Wilcoxon Mann–Whitney test with Bonferroni correction).

The detection of prey by *P. pacificus* predators likely acts through ciliated sensory neurons in its head (Moreno et al. 2019) and with our findings revealing that TGF-β signaling is essential for aspects of environmental sensing in *P. pacificus*, we next investigated if predatory behaviors were also disrupted in these mutants (fig. 4*A*). Using previously established predation assays (Wilecki et al. 2015), we first investigated if the detection of prey was abrogated in TGF-β signaling mutants. Only *Ppa-tag-68* mutants showed statistically significant defects (*P* = 0.006, Wilcoxon Mann–Whitney test with Bonferroni correction) in detecting *C. elegans* prey (fig. 4*B*). Mutations in *Ppa-tag-68* also had the strongest effect on mouth formation (fig. 3*B*). However, due to the pleiotropic and sickly nature of *Ppa-tag-68* mutants, it is difficult to validate if this is caused by a direct defect in prey detection in this mutant or from some of the other defects observed.

**Fig. 5.**
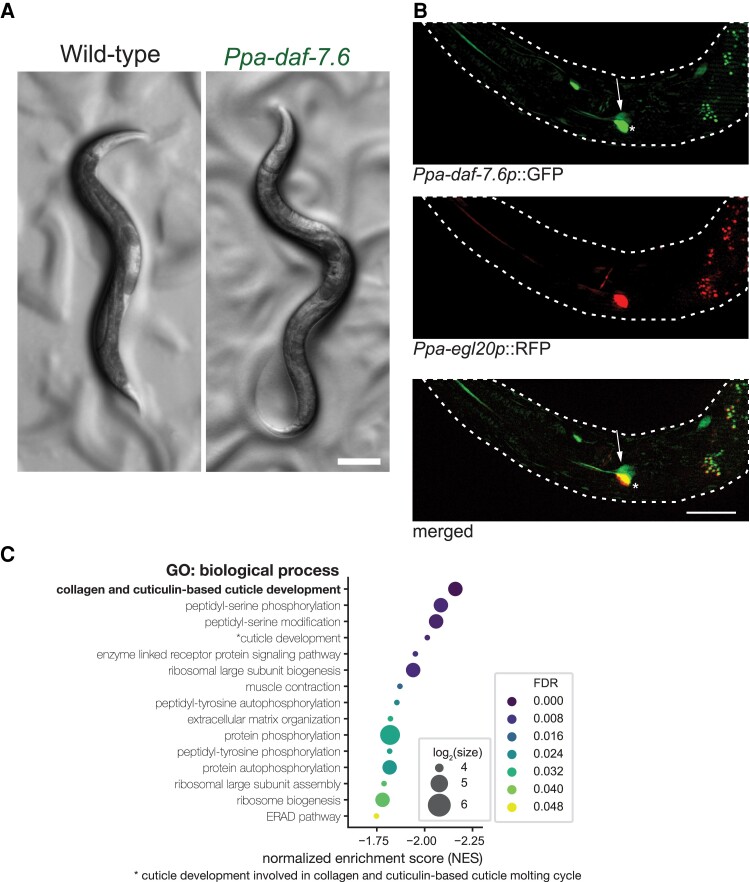
*Ppa-daf-7.6* influences cuticle integrity. (*A*) Some *Ppa-daf-7* animals have an abnormal body morphology. Scale bar = 100 µm. (*B*) Expression pattern of *Ppa-daf-7.6p*::GFP. The strongest and most consistent expression was detected in cell “*” which overlaps with AM5(ASE), expressed by the co-injection marker *Ppa-egl-20p*::RFP. The GFP expressing neuron dorsal to the AM5 is likely the AM4(ASK) amphid neuron (arrow). Scale = 10 µm. (*C*) Plots showing normalized enrichment scores of the gene set enrichment analysis in GO biologic process of *Ppa-daf-7.6*. The size indicates the number of genes in each gene set. Collagen- and cuticle-associated gene expression is most affected by mutations in *Ppa-daf-7.6.* FDR, false discovery rate.

In addition to the predation of other nematodes, *P. pacificus* also engages in cannibalistic behaviors of other con-specifics; however, it does not kill its own progeny nor close relatives due to the presence of a kin-recognition system dependent on a small peptide encoded by *self-1* (Lightfoot et al. 2019; Lightfoot et al. 2021). Therefore, we explored if TGF-β signaling was also involved in the establishment of the kin-recognition system in *P. pacificus*. Using TGF-β signaling mutants as predators, we probed for any defects in the recognition of the kin-signal on larvae. However, no defects were observed and all mutants avoided killing their kin (fig. 4*C*). Next, we investigated the TGF-β signaling mutants for failures in the formation of the kin-signal by using the TGF-β signaling mutants as prey (fig. 4*D*). One TGF-β mutant, *Ppa-daf-7.6*, showed a significant defect in the kin-recognition signal (*P* = 0.003, Wilcoxon Mann–Whitney test with Bonferroni correction) and was more likely to be erroneously killed by *P. pacificus* predators. In *Ppa-daf-7.6* mutants, a proportion of animals have an abnormal body morphology which, while not fully penetrant, is particularly evident early in development (fig. 5*A* and supplementary fig. S3, Supplementary Material online). *Ppa-daf-7.6* is expressed in a pair of amphid sensory neurons in the head identified as AM5, which corresponds to the ASE neuron in *C. elegans* (Hong et al. 2019). In addition, we also observed expression in another amphid neuron pair likely to be the AM4(ASK) amphid neuron (fig. 5*B*). Subsequently, to investigate the kin-recognition defect observed in *Ppa-daf-7.6* further, we utilized RNA-Seq. Although we observed no change in *self-1* expression levels, a significant reduction in the expression of genes responsible for collagen and cuticle development was detected in this mutant (fig. 5*C* and supplementary fig. S6, Supplementary Material online). As an intact cuticle is essential for the kin-recognition signal (Lightfoot et al. 2019), we hypothesize that the kin-recognition defects may result from errors in cuticle formation which in turn may lead to the mis-identification of kin by predators resulting in there killing.

## Discussion

Here, we explored TGF-β signaling across nematode evolution with an emphasis on functional studies in *P. pacificus* and revealed numerous conserved and divergent aspects to this pathway (summarized in supplementary table S3, Supplementary Material online). Although we firstly demonstrated a conserved function in regulating body morphology in the *Ppa-dbl-1* associated pathway, other TGF-β ligand pathways regulate diverse functions distinct from those described in *C. elegans*. This includes the *Ppa-unc-129* signaling ligand which shows no *unc* phenotype in *P. pacificus* and is unlikely to share the role in axon guidance described in *C. elegans*. Additionally, the *Ppa-tig-2* pathway appears to have a role in establishing the correct body size in *P. pacificus*, whereas in *C. elegans*, it is also associated with neuronal guidance (Baltaci et al. 2022). However, the most striking differences between species were observed in the DAF-7 signaling network. This also coincides with a significant variation in copy number in DAF-7-associated genes. In *P. pacificus*, seven copies of the *Ppa-daf-7* ligand were detected as well as three copies of the type II receptor *Ppa-daf-4* and four copies of the Co-Smad *Ppa-daf-3*, resulting in the potential for a degree of functional redundancy. Moreover, our functional analysis of these genes led to one of the most surprising findings as although these genes are canonically associated with the regulation of the dauer life cycle stage in *C. elegans*, this function may be absent in *P. pacificus*. As the dauer stage is often thought homologous to the infective larvae of parasitic nematodes, the role of TGF-β in dauer regulation has been the focus of much parasitic research (Viney et al. 2005). Indeed, in several parasitic nematode species, TGF-β expression is higher during the infective larval stage (Gomez-Escobar et al. 2000; Crook et al. 2005; Stoltzfus et al. 2012); however, functional experiments have failed to confirm its significance for infective larval formation. This includes studies investigating *daf-7* in *Parastrongyloides trichosuri* which failed to complement *C. elegans daf-7* mutants (Crook et al. 2010) reinforcing our own functional results. Thus, TGF-β may have been co-opted for dauer regulation at some point during the evolutionary lineage leading to *C. elegans* but its involvement in dauer formation may not be a ubiquitous phenomenon conserved across nematodes. This prediction will require additional experimental validation via the creation of a further *Ppa-daf-7* septuple mutant as well as functional studies in other nematode species. Importantly, this may have repercussions for parasitic nematode research as well as our understanding of the evolution of nematode parasitism in general.

In addition to dauer formation, the DAF-7 pathway has also been shown to be associated with other environmental sensing phenotypes in *C. elegans* (de Bono et al. 2002; Nolan et al. 2002; Meisel et al. 2014). Although our assessment of sensory defects in *P. pacificus* is far from exhaustive, our experiments demonstrate that chemosensory defects are also evident in *Ppa-daf-7* mutants. This includes deficiencies in the response of *P. pacificus* to an insect associated pheromone cue observed in the *Ppa-daf-7.1; Ppa-daf-7.2* and a shift in its mouth-form frequency. However, as sensing was only disrupted in mutants involving double knockouts, it is likely that there is a high degree of redundancy in their function. This is in stark contrast to *C. elegans* in which only a single *Cel-daf-7* homologue exists. Within the *Ppa-daf-7* signaling system, we also identified an important role for the *Ppa-daf-7.6* paralogue in regulating the kin-recognition signal. A transcriptome analysis of *Ppa-daf-7.6* revealed reduced expression in collagen and cuticle development genes which are also transcriptional targets of TGF-β signaling in *C. elegans* (Goodman and Savage-Dunn 2022). Thus, we hypothesize that in *Ppa-daf-7.6*, integrity of the cuticle is compromised which in turn interferes with the establishment of the kin-signal causing kin-recognition defects (Lightfoot et al. 2019). Future studies therefore, must seek to explore *Ppa-daf-7* further by investigating expression patterns across the paralogues as well as additional alleles with diverse mutations in order to understand if different receptor families are established in different sensory neurons and their individual functional contributions.

In addition to differences in the TGF-β signaling ligands, there are also striking phenotypic differences between species in the I-Smad, *tag-68*. In *Cel-tag-68*, no phenotypes have been reported; however, *Ppa-tag-68* is associated with a multitude of defects resulting in sickly, slow growing animals. These are caused by deficiencies in cellular pathways regulating DNA processing, lipid storage, transcription and protein synthesis, as well as errors in developmental processes resulting in a small body size and the altering of the mouth-form frequency. In other species, I-Smads are known to regulate both Smad and non-Smad pathways in order to adjust TGF-β signaling levels and are associated with numerous clinical diseases in humans (Miyazawa and Miyazono 2017). With only a single I-Smad homologue identified in both nematode species, it is therefore somewhat surprising that there is no reported phenotype in *Cel-tag-68* mutants. Our findings in *P. pacificus* may therefore help elucidated the mechanisms of I-Smad function in nematodes and in other species. Thus, our work demonstrates a previously unknown and surprising flexibility in the TGF-β signaling pathways across nematodes. This coincides with the evolution and regulation of diverse developmental and behavioral traits as distinct as dauer formation in *C. elegans* and mouth-form structure in *P. pacificus*, and reinforces the importance of exploring signaling networks outside of canonical model species to reveal fresh insights into their function.

## Materials and Methods

### Nematode Husbandry

All nematodes used were maintained on standard NGM plates and maintained on a diet of *Escherichia coli* OP50. All strains used in this study can be found in supplementary table S1, Supplementary Material online.

### Identification of Orthologous of TGF-β Genes and Construction of Gene Tree

We collected published genome assemblies of nine nematode species representing four of the five major nematode clades (Blaxter et al. 1998) *C. elegans* (WormBase website [https://wormbase.org], release WS271 2019), *H. contortus* (Laing et al. 2013)*, P. pacificus* (Rödelsperger et al. 2017), *Strongyloides ratti* (Kikuchi et al. 2011; Hunt et al. 2016), *Brugia malayi* (Choi et al. 2011; Foster et al. 2020), *Ascaris suum* (Wang et al. 2017), *Romanomermis culicivorax* (Schiffer et al. 2013), and *Trichinella spiralis* (Korhonen et al. 2016). The orthologous of TGF-β genes was identified using OrthoFinder (Emms and Kelly 2015, 2019). To interpret the evolutionary history of *daf-7* genes in the genus *Pristionchus*, we identified the orthologous of *daf-7* genes based on the published *Pristionchus* genomes (Prabh et al. 2018). The *daf-7* genes were aligned by MUSCLE (Edgar 2004) and the gene tree was constructed by PhyML (Guindon et al. 2010).

### CRISPR/Cas9-Induced Mutations

Mutations were induced in candidate genes via CRISPR/Cas9. Gene-specific crRNA and universal trans-activating CRISPR RNA (tracrRNA) was purchased from Integrated DNA Technologies and 5 μl of each 100 μM stock mixed and denatured at 95 °C for 5 min and allowed to cool down to room temperature to anneal. Cas9 was added to the hybridized product with Cas9 added to the mix (NEB or Integrated DNA Technologies) and incubated at room temperature for 5 min. This was subsequently diluted with TE buffer to a final concentration of 18.1 μM for the sgRNA and 2.5 μM Cas9. This was injected into the germline of *P. pacificus* strain PS312. Eggs from injected P0s were recovered up to 16 h post injection. After hatching and 2 days’ growth, these F1 were segregated onto individual plates until they had also developed sufficiently and egg-laying had been initiated. The genotype of the F1 animals was subsequently analyzed via Sanger sequencing and mutations identified before re-isolation in homozygosis. sgRNAs and associated primers utilized in this study can be found in supplementary table S2, Supplementary Material online. Evidence for the generation of putative null mutants was based upon frame shifts leading to premature stop codons in the protein coding sequences and additionally we utilized NetGene2 which failed to identify any alternative splice sites in these mutants.

### Mutant Size Analysis

Worms were first synchronized by bleaching well-populated plates containing an abundance of young adults to acquire egg cultures. These were washed three times with M9 to remove bleach, maintained in M9 overnight and allowed to hatch in the solution. Newly hatched larvae were filtered through two 20 µm filters to remove carcases and transferred to OP50 seeded NGM plates. Worms were imaged at time points 0, 24, 48, and 72 h post transfer to the NGM plate. Worms were imaged on a Zeiss Axio Zoom v.16 and worm size was measured using the Wormsizer plugin for Fiji/ImageJ (Moore et al. 2013).

### Dauer Assay

*Pristionchus pacificus* wild-type PS312 grown in laboratory conditions enter dauer upon crowding and starvation conditions. To determine a possible dauer-constitutive phenotype (*daf-c*), we initiated cultures with ten young adult gravid hermaphrodites. The *Ppa-hsd-2* mutant was used as a *daf-c* positive control and as it forms more dauers at 25 °C all mutants were assessed at both 20 °C and 25 °C. For the 20 °C cultures, we looked for dauer larvae on days 6–10, just prior to starvation. For the 25 °C cultures, we looked for dauer larvae on days 4–6, also just prior to starvation. After starvation, we used the same cultures to look for the absence of dauer larvae on days 10–14 as indications of possible dauer formation defects (*daf-d*). We counted the number of dauer larvae and J3 larvae (dauer equivalent) for up to 100 total nematodes for each culture. We observed at least three cultures for each strain at both temperatures.

### Chemosensation Assay

Nematodes were cultured at 20 °C on OP50 seeded NGM plates until just before food was depleted. After washing twice with M9 buffer, ∼100 adult hermaphrodites were loaded onto the middle edge of 6 cm plates containing agar as previously described (10 mM MOPS pH 7; 2.5% Tween 20; 1.5% Bacto-agar; Nuttley et al. 2002; Margie et al. 2013; Carstensen et al. 2021). About 1.5 µl of 2 M sodium azide was spotted on opposing sides, followed by adding 1.5 µl of the attractive odor or 99% ethanol control. Each assay involved at least 10 animals in the scoring arena and each condition was the summary of at least three experimental sessions totaling 12–15 assays, with the exception of the *Ppa-hsd-2(csu60)* control for enhanced odor attraction, of which only 9 assays were performed. Chemotaxis assays lasted 15–20 h with the exception of *Ppa-daf-7.1* and *Ppa-daf-7.2* which were scored after 6–9 h due to their more rapid dispersal. MM was purchased from Sigma-Aldrich (St Louis, MO, USA) and diluted with 99% ethanol.

### Aggregation and Bordering

Larvae were passed through two 20 µm filters to isolated synchronized J2 larvae and put onto OP50 seeded NGM plates. The following day, assay plates were prepared by dropping 50 µl of OP50 into the center of an NGM plate in order to form one small lawn of bacteria and let at room temperature to ensure bacterial growth. Three days following the filtration, worms were retrieved from plates in 3 ml M9 and rinsed with additional M9 to remove excess bacteria from them. After centrifugation, worms were left on unseeded NGM plates for 1 h. Sixty worms were then picked and distributed around the bacterial lawn of assay plates and put at 20 °C. After 3 h, plates were imaged using a Zeiss Axio Zoom v.16 at 12.5× magnification and the position of worms was assessed using the cell counter plugin for Fiji/ImageJ. Clumping was assessed as the ratio of animals found on the bacterial lawn in contact with another individual. Bordering was assessed as the ratio of animals found at the border of the bacterial lawn.

### Mouth Form

Prior to mouth-form phenotyping, all mutants were grown for three generations consecutively on NGM plates with OP50 after which the mouth-form phenotyping was attained through observation of the nematode buccal cavity using a Zeiss SteREO Discovery V12 microscope. Morph identities were categorized based on previous described species characteristics and could be subsequently verified by mounting and 40× Nomarski examination. Final mouth-form frequencies are the mean of five independent replicates. Statistics were carried out based on previous methods (Lightfoot et al. 2021)

### Lipid Staining

Oil Red O was utilized to visualize the nematode internal lipid abundance and distribution according to previous studies (Escorcia et al. 2018). Briefly, an Oil Red O stock solution was made by dissolving 500 mg Oil Red O powder in 100 ml of 100% isopropanol and maintained in the dark. From this, a working solution was made by diluting the stock in water to a final concentration of 60% Oil Red O and was passed through a 0.22 µm filter before use. Worms were transferred to a microcentrifuge tube using PBSTriton (0.01%) and subsequently washed three further times in PBSTriton to remove excess bacteria. Supernatant and worms suspended in 100 µl PBSTriton. About 600 µl 40% isopropanol was added to the worm pellet and rocked at room temperature for 3 min. Worms were then centrifuged at 560 × *g* for 30 s and supernatant 600 µl of supernatant removed leaving them in 100 µl solution. About 600 µl Oil Red O working solution was added to each sample and mixed well by rotating for 2 h at room temperature. Samples were centrifuged at 560 × *g* for 1 min and 600 µl solution removed. A further 600 µl of PBSTriton was then added and the samples mixed by rotating for 30 min to removed excess Oil Red O. Samples were then centrifuged at 560 × *g* for 1 min and all but 50 µl of supernatant removed. Worms were then resuspended in the remaining solution and 5 µl of worm suspension placed onto a microscope slide and a coverslip added on top. Worms were then imaged immediately on a Nikon E1000 microscope and a Bresser MikroCam II 12 MP color camera. The brightness of the gut was averaged from three areas of 23.68 µm and normalized by the intensity in the mouth which shows no staining. The inverse of the resulting intensity was plotted.

### Generating Transgenes

Transcriptional reporter lines were generated by polymerase chain reaction (PCR) amplification of a 1.5 kb region upstream of both target genes, *Ppa-tag-58* and *Ppa-daf-7.6* which was predicted to contain their promoter elements. Subsequently, this was cloned into a pUC19 vector containing the codon optimized GFP or TurboRFP and Ppa-rpl-23 3′UTR with *P. pacificus* introns (Han et al. 2020). Stable lines were generated by digesting and linearizing the plasmids with PstI and 10 ng/ml of this was subsequently co-injected into the *P. pacificus* germline along with 60 ng/ml genomic carrier DNA, cut with PstI, and for *Ppa-daf-7.6*, 10 ng/ml of the co-injection marker *Ppa-egl-20p*::RFP also cut with PstI. The same expression pattern was confirmed in multiple independent lines. Individual animals were imaged using a Zeiss AxioImager Z1 microscope and Axiocam506 mono camera. Primers used to generate transgenic lines can be found in supplementary table S4, Supplementary Material online.

### Predation and Kin-recognition Assays

Corpse assays facilitated rapid quantification of predatory behavior allowing us to test predation and kin-recognition across the large number of mutants. Prey was maintained on NGM plates seeded with OP50 bacteria until freshly starved, resulting in an abundance of young larvae. These plates were washed with M9 and passed through two 20 µm filters to isolate pure cultures of larvae. They were subsequently centrifuged before being deposited on to an unseeded assay plate by pipetting 1 µl of *C. elegans* larval pellet or 1.5 µl of *P. pacificus* larval pellet on to a 6 cm NGM unseeded plate. Five predatory nematodes were screened for the appropriate mouth morph and added to assay plates for *C. elegans* prey assays. They were then permitted to feed on the prey for 2 h and the plate was subsequently screened for the presence of corpses. When utilizing *P. pacificus* as prey for kin-recognition assays, 20 predators were placed on assays and left for 24 h before the plate was subsequently screened for the presence of corpses.

### Statistics

Statistical tests were performed using the R software. Box plot represents the distribution of the data using quartiles (Q). Q1, Q2, and Q3 being, respectively, the values below which lie 25%, 50%, and 75% of the data points. Q1 and Q3 form the limits of the box and the median Q2 is indicated by a bolder line. The whiskers represent the range in which most values are found, whereas the values outside represent the outliers (*P ≤ 0.05, **P ≤ 0.01, ***P ≤ 0.001, and ****P ≤ 0.0001).

### RNA Sequencing and Data Analysis

Mixed-staged *P. pacificus* animals were collected from three NGM plates and were washed three times using M9 buffer. Total RNA was extracted using Direct-Zol RNA Mini prep kit (Zymo Research) according to the manufacturer's guidelines. The RNA-seq library preparation and sequencing were done by the company Novogene. Raw reads have been uploaded to the Sequence Read Archive under the BioProject ID PRJNA828394. Software Hisat2 (version 2.1.0; Kim et al. 2015) was used to map raw reads to the *P. pacificus* reference genome (pristionchus.org, version: El Paco [Rödelsperger et al. 2017], featureCounts [Liao et al. 2014]) was used to quantify the counts of reads mapped to the genomic feature based on the El Paco gene annotations V3. The biologic pathways that are differentially expressed were detected using Gene Set Enrichment Analysis (Subramanian et al. 2005). The collection of gene sets was obtained from WormEnrichr (Kuleshov et al. 2016), and the genes of *P. pacificus* were assigned into corresponded categories based on their orthology with *C. elegans*.

### qRT-PCR

Wild-type, *Ppa-daf-7.6*, and *Ppa-tag-68* animals were first synchronized by bleaching and allowed to hatch in M9 buffer for 24 h. Animals were collected at mid-J4, late J4, and young adult stages. RNA extraction was performed as described above. qPCR assays were performed using LightCycler 480 Instrument II (Roche Life Science) and iTaq Universal SYBR Green One-Step Kit (Bio-Rad Laboratories). We used *Ppa-gpd-3*, *Ppa-eif-3*, and *Ppa-csq-1* genes as internal controls for relative quantification of gene expression. The relative-fold changes were calculated using the ΔCT method. Primers used can be found in supplementary table S4, Supplementary Material online.

### Identification of the Oscillating Genes

Temporally highly resolved RNA-seq data of *P. pacificus* were obtained from our previous study (Sun et al. 2021). The RNA-seq analysis was performed as described above. The expression patterns of gene sets that were differentially expressed were clustered by hierarchically clustered heatmap to identify the subset of the oscillating genes.

## Supplementary Material

msac252_Supplementary_Data

## Data Availability

All strain information is available in supplementary table S1 and all strains are available upon request. In addition, RNA-sequence data is available from the Sequence Read Archive under the BioProject ID PRJNA828394.
