# Supplementary material for: Evolution and Diversity of TGF-β Pathways are Linked with Novel Developmental and Behavioral Traits
Source: Mol Biol Evol. 2022 Dec 5;39(12):msac252. doi: 10.1093/molbev/msac252 (PMC9733428; doi:10.1093/molbev/msac252)

**SFigure 1.** An illustration of TGF-β gene structures in *P. pacificus* and CRISPR/Cas 9 knockout sites (dark green line).

**SFigure 2.** (A) Genomic sequences (grey) and amino acids (green) of the wild-type and sequencing results of mutations induced by CRISPR/Cas9. Red rectangles indicate the protospacer adjacent motif (PAM) targeted for cleavage by the CRISPR-Cas9. Top row is WT sequence, lower row shows mutant sequence. (B) The screenshots of the Integrative Genomics Viewer show RNA-seq mapping results. In *Ppa-daf-7.6* and *Ppa-tag-68* mutants, no raw read was mapped to the deletion region, indicating these two are homozygous mutations.

**SFigure 3.** TGF-β mutants show morphological size defects throughout development. (A) newly hatched J2 larvae (B) 24 hours post hatching (C) 48 hours post hatching. N= at least 22 animals for all mutants analysed. (Wilcoxon Mann Whitney test with Bonferroni correction (non-significant (ns), p-value ≤ 0.05 (*), ≤ 0.01 (**), ≤ 0.001 (***), ≤ 0.0001 (****)).

**SFigure 4.** *Ppa-daf-7* homologs are expressed in J2 (top) and dauer (bottom) larvae. In addition to expression in adult hermaphrodites, *Ppa-daf-7.1p::*GFP (left); *Ppa-daf-7.2p::*RFP(middle) reporters are expressed in amphid neurons in well-fed J2 (n=42) and starvation-induced dauer larvae (n=25). Overlay (right) of a dauer shows pronounced amphid opening and constricted pharynx characterizing the *P. pacificus* dauer stage. Anterior is left and dorsal is top. Scale bar = 20 μm.

**SFigure 5.** Aggregation behaviours in *Ppa-daf-7* TGF-β mutants. (A) Ratio of mutant animals found aggregating in clumps compared to Wildtype and RSB001 controls. (B) Ratio of mutant animals found bordering compared to Wildtype and RSB001 controls. RSB001 is found from a high-altitude environment and is known to form clumps and border extensively under laboratory conditions. N = 5, Wilcoxon Mann Whitney test with Bonferroni correction (non-significant (ns), p-value ≤ 0.05 (*)). (C) Representative images of all the TGF-β mutants analysed for aggregation behaviours. Scale bar = 1000 μm.

**SFigure 6** Transcriptomic profiling of *Ppa-daf-7.6* and *Ppa-tag-68* (A) Expression profiles of mixed-stages mutant animals with stage-specific wild-type animals of gene sets associated with cuticle development (GO:0040002 and GO:0042338) in *Ppa-daf-7.6* and *Ppa-tag-68* and fatty acid beta-oxidation (GO:0006635) in *Ppa-tag-68.* (B) Temporally highly resolved transcriptomic profile of *P. pacificus.* Subsets of genes of cuticle development categories (GO:0040002 and GO:0042338) show oscillatory patterns as indicated by vertical black lines

on the left side. The expression profile of the fatty acid beta-oxidation (GO:0006635) category is an example of genes that do not have an oscillatory pattern. (C) qRT-PCR results of five oscillating genes (highlighted by the bold font in B). The expression patterns of wild-type, *Ppa-daf-7.6*, and *Ppa-tag-68* at middle J4, late J4, and young adult stages (triangles at panel B) were measured. $p$-value < 0.05 (*).

**Supplementary Movie 1.** *Ppa-unc-129* (right) shows no uncoordinated movement and is wild-type in appearance and motility. Wild type animal also shown for comparison (left).

**Supplementary table S1.** List of all genes and alleles used in this study.

**Supplementary table S2.** Gene location**,** sgRNAs and associated primers utilised in this study.

**Supplementary table S3.** Comparative summary table of phenotypes associated with the TGF-β signalling pathway in *C. elegans* and *P. pacificus*. Function attributed by this study are indicated in black.

**Supplementary table S4.** List of primers used to generate transgenic lines and qPCR analysis.

Supplementary fig. S1

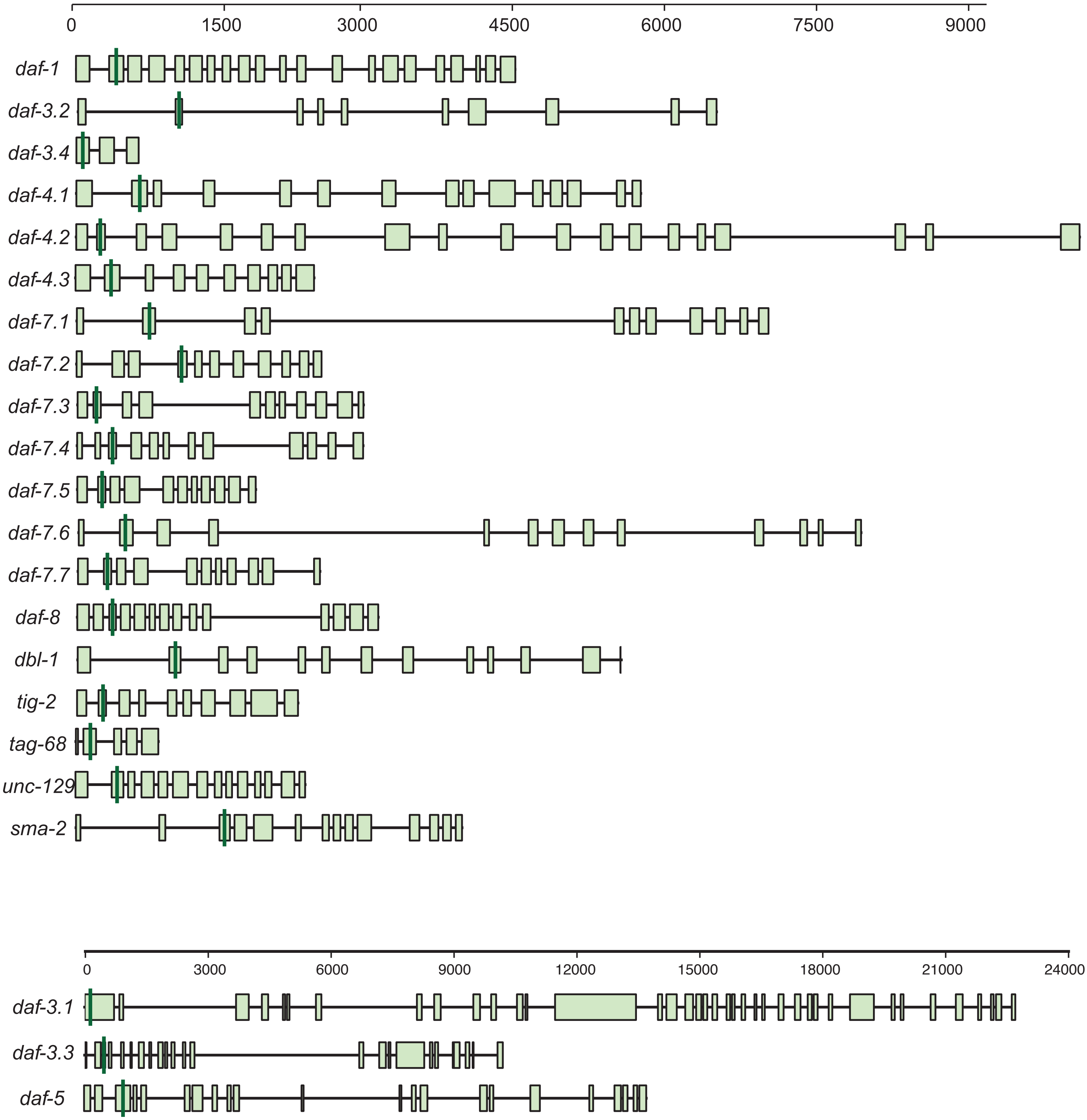

Supplementary fig. S2

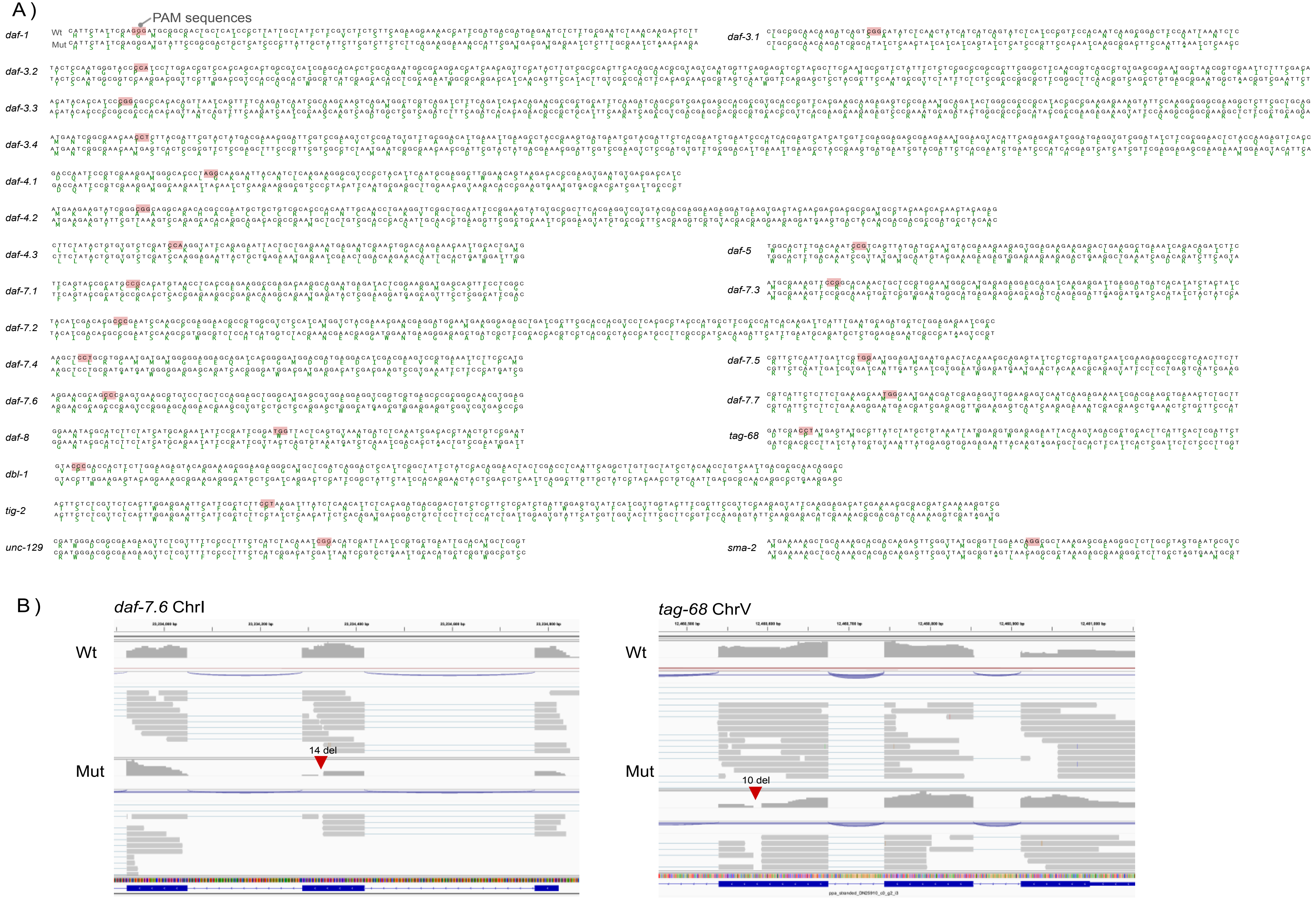

Supplementary fig. S3

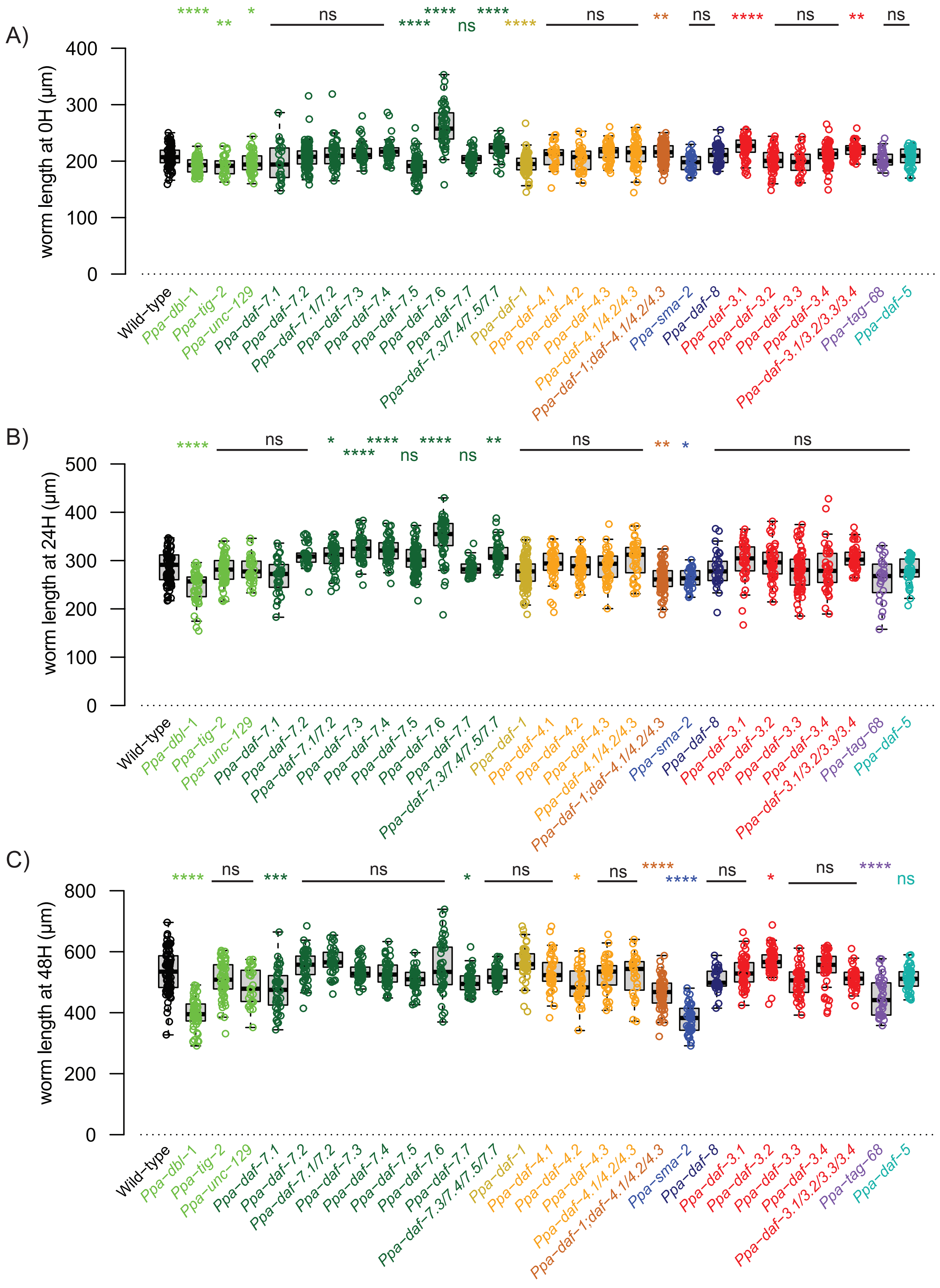

Supplementary fig. S4

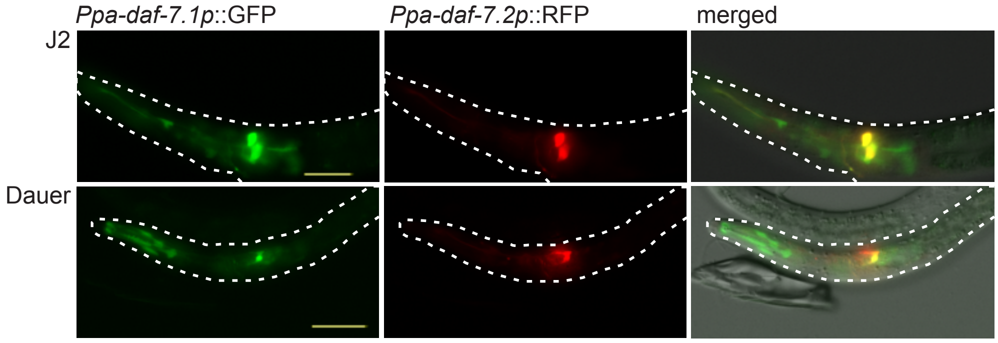

Supplementary fig. S5

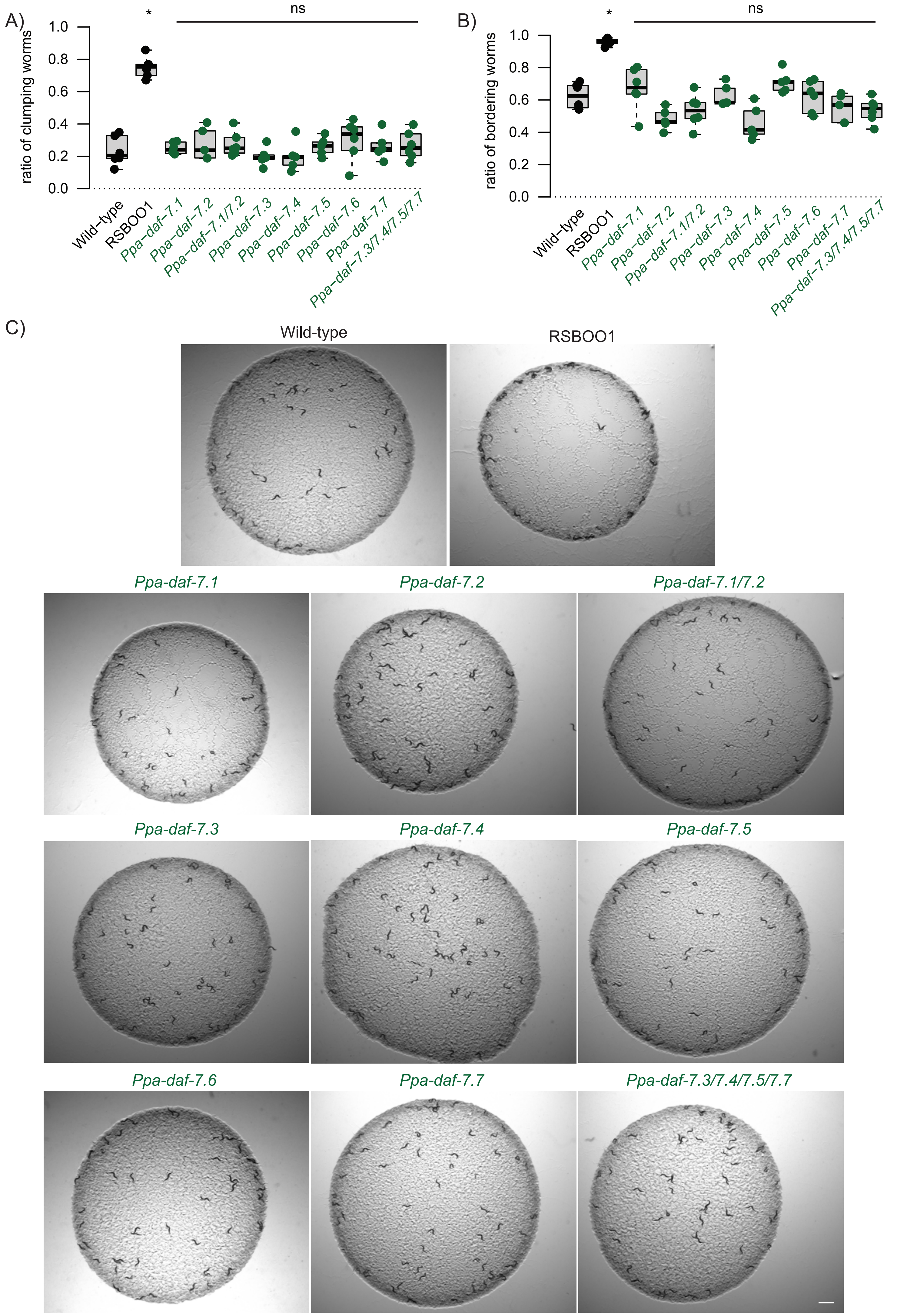

Supplementary fig. S6

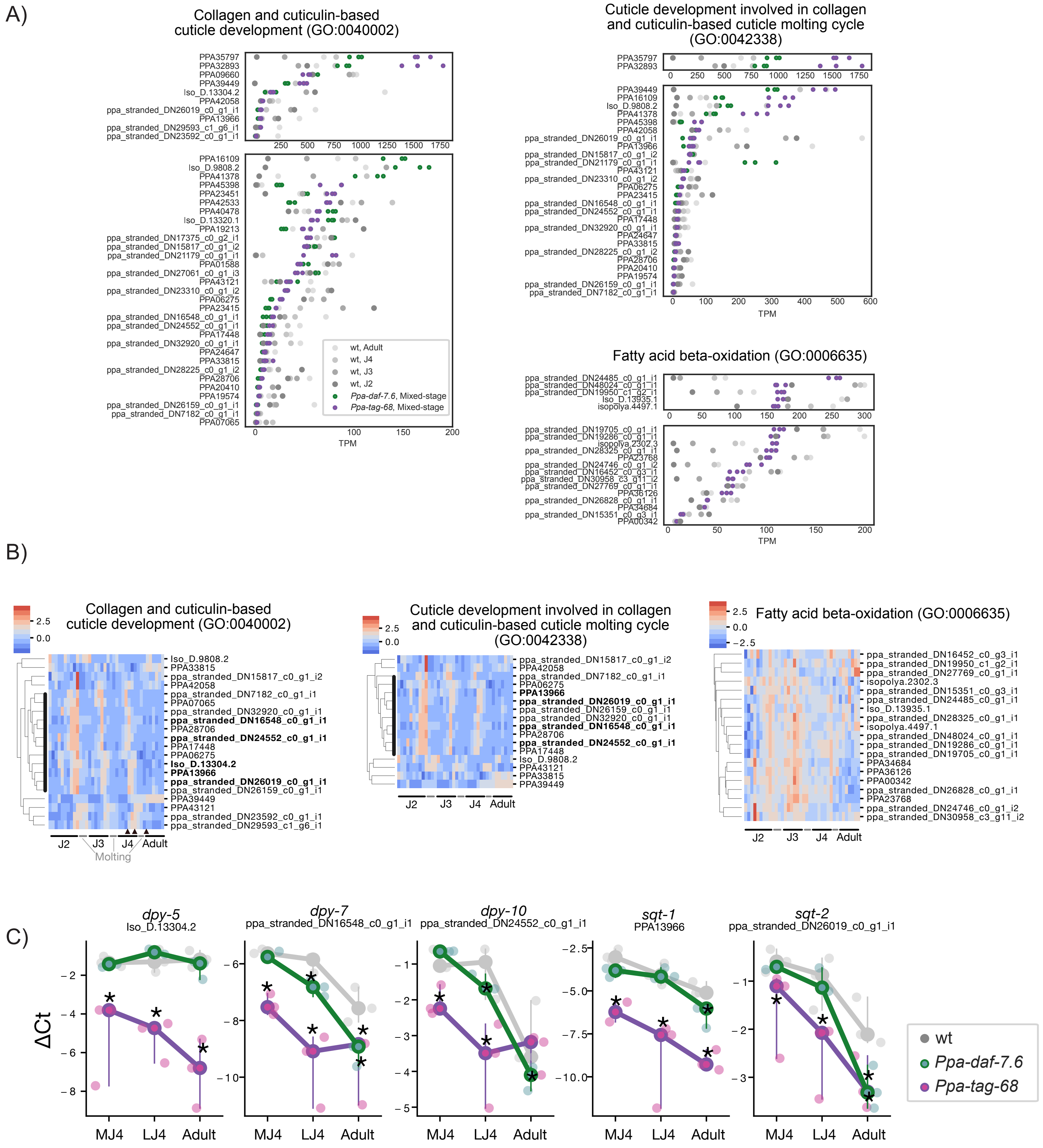

Supplement: msac252_Supplementary_Data [file msac252_supplementary_data.zip › Supplementary_Figs.pdf]
